# Supplementary material for: Synthetic ShK-like Peptide from the Jellyfish Nemopilema nomurai Has Human Voltage-Gated Potassium-Channel-Blocking Activity
Source: Mar Drugs. 2024 May 13;22(5):217. doi: 10.3390/md22050217 (PMC11122761; doi:10.3390/md22050217)
Supplement: Supplementary file 1 [file marinedrugs-22-00217-s001.zip › Figure legneds.pdf]

Figure legends

Figure S1. Results from Pepmic Co. Ltd. for the purification of synthetic NnK-1 using high-performance liquid chromatography.

Figure S2. Mass spectrometry analysis conducted by Pepmic Co. Ltd. illustrating the results subsequent to the formation of the first pair of disulfide bonds.

Figure S3. Mass spectrometry analysis conducted by Pepmic Co. Ltd. illustrating the results subsequent to the formation of the second pair of disulfide bonds.

Figure S4. Mass spectrometry analysis conducted by Pepmic Co. Ltd. illustrating the results subsequent to the formation of the third pair of disulfide bonds.
