# Supplementary material for: Synthetic ShK-like Peptide from the Jellyfish Nemopilema nomurai Has Human Voltage-Gated Potassium-Channel-Blocking Activity
Source: Mar Drugs. 2024 May 13;22(5):217. doi: 10.3390/md22050217 (PMC11122761; doi:10.3390/md22050217)
Supplement: Supplementary file 1 [file marinedrugs-22-00217-s001.zip › Figure S3.pdf]

MS Spectrum

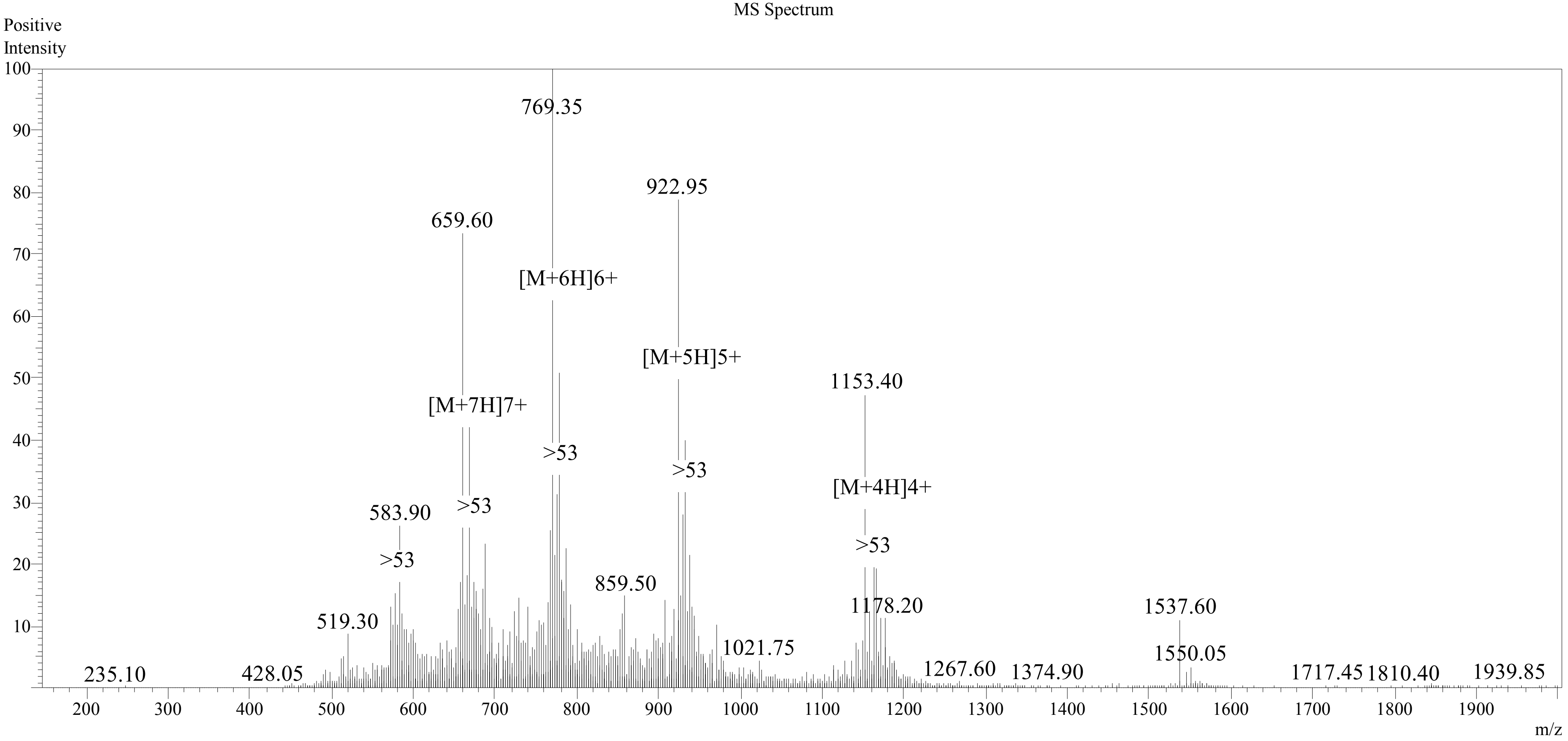

Sample Information		Interface	:ESI	Prerod Bias	:+4.5kv
Dissolution method	:3%HAC+25%ACN+72%H2O	Nebulizing Gas Flow	:1.50L/min	Detector	:-0.2kv
Date Acquired	: 2019/12/3 14:53:45	CDL Temp	:250C	T.Flow	:0.2ml/min
Injection Volume	: 1ul	CDL Volt	:0v	B.conc	:30%H2O/70%MeOH
		Block Temp	:200		

Name	:g5156.t1-3
Sequence	:*CKDHHTYGVY*CKDWKSSGECKKNPKGMRHF*CRKTCGF*C
Lot No	:PCM14777-1-1023
Theoretical	:4610.24
Observed	:4610.10
