# Supplementary figures and images for: Synthetic ShK-like Peptide from the Jellyfish Nemopilema nomurai Has Human Voltage-Gated Potassium-Channel-Blocking Activity
Source: Mar Drugs. 2024 May 13;22(5):217. doi: 10.3390/md22050217 (PMC11122761; doi:10.3390/md22050217)

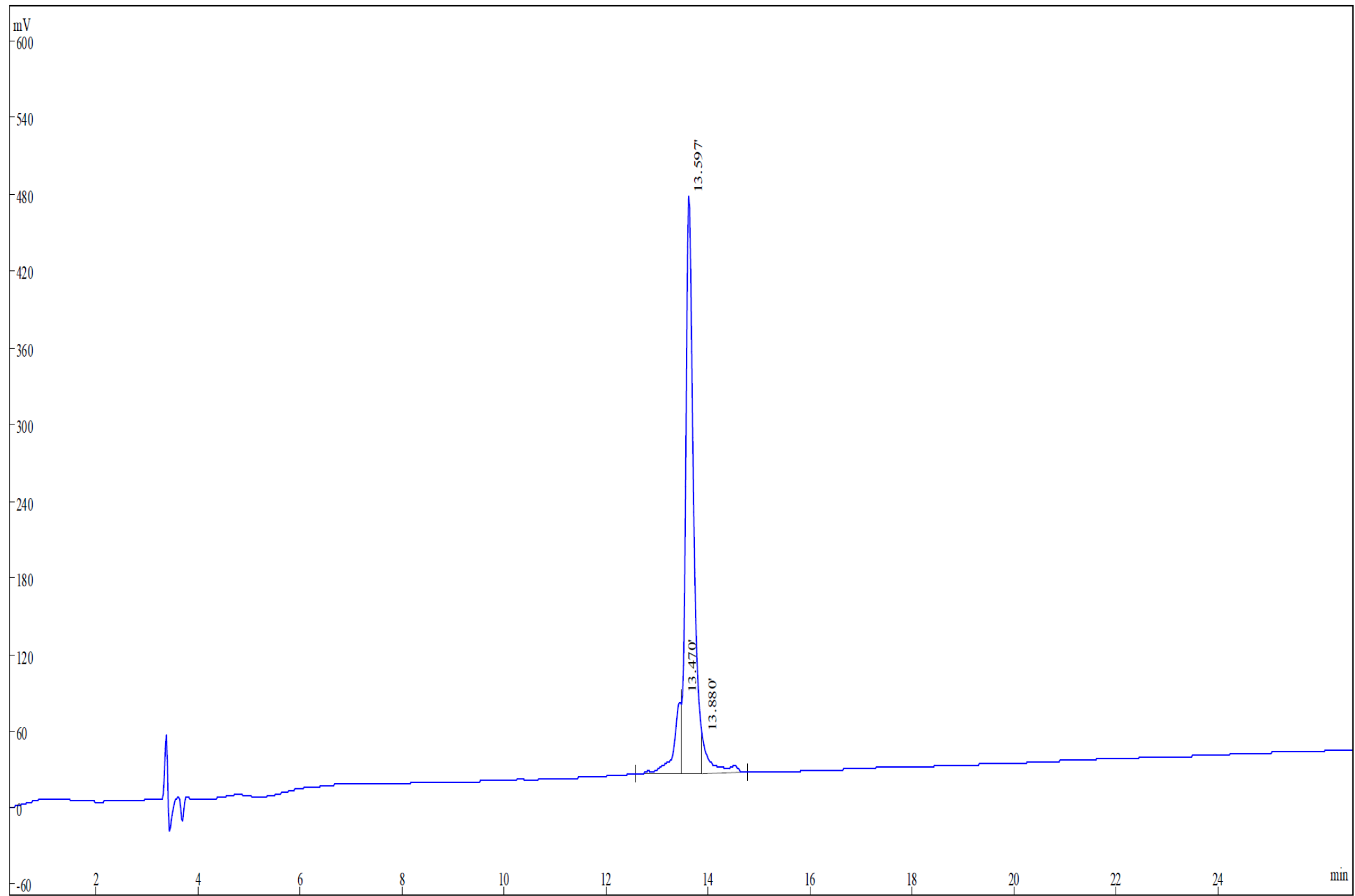

Supplement: Supplementary file 1 [file marinedrugs-22-00217-s001.zip › Figure S1.pdf]
